# Application of EST-SSR markers developed from the transcriptome of *Torreya grandis* (Taxaceae), a threatened nut-yielding conifer tree

**DOI:** 10.7717/peerj.5606

**Published:** 2018-09-19

**Authors:** Jun Zeng, Jie Chen, Yixuan Kou, Yujin Wang

**Affiliations:** 1 Institute of Tropical Bioscience and Biotechnology, Chinese Academy of Tropical Agricultural Sciences, Haikou, China; 2 State Key Laboratory of Grassland Agro-Ecosystems, School of Life Sciences, Lanzhou University, Lanzhou, China; 3 Laboratory of Subtropical Biodiversity, School of Agricultural Sciences, Jiangxi Agricultural University, Nanchang, China

**Keywords:** *Torreya grandis*, Transcriptome, EST-SSRs, Population structure

## Abstract

*Torreya grandis* (Taxaceae) is an ancient conifer species endemic to southeast China. Because of its nutrient-rich and delicious seeds, this species has been utilized for centuries by the Chinese. However, transcriptome data and transcriptome-derived microsatellite markers for population genetics studies are still insufficient for understanding of this species’ genetic basis. In this study, a transcriptome from *T. grandis* leaves was generated using Illumina sequencing. A total of 69,920 unigenes were generated after de novo assembly, and annotated by searching against seven protein databases. In addition, 2,065 expressed sequence tag–simple sequence repeats (EST-SSRs) were detected, with the distribution frequency of 2.75% of total unigenes and average number of 0.03 SSRs per unigene. Among these EST-SSRs, 1,339 primer pairs were successfully designed, and 106 primer pairs were randomly selected for the development of potential molecular markers. Among them, 11 EST-SSR markers revealed a moderate level of genetic diversity, and were used to investigate the population structure of *T. grandis*. Two different genetic groups within this species were revealed using these EST-SSR markers, indicating that these markers developed in this study can be effectively applied to the population genetic analysis of *T. grandis*.

## Introduction

The Chinese nutmeg tree, *Torreya grandis* Fort. et Lindl. (Taxaceae), is a conifer species restricted to only a few mountainous regions in Zhejiang, Anhui, Jiangxi, Hunan, and Fujian, China (Kang & Tang, 1995; Cheng et al., 2007). Its seeds, well-known as a nutrient-rich delicacy, have been utilized by Chinese people for centuries (Kang & Tang, 1995; Li et al., 2005; Cheng et al., 2007). Moreover, its wood, being highly valued for its durability, along with pest and decay resistance, was widely used to make furniture, resulting in overexploitation (Yang & Luscombe, 2013). In combination with habitat destruction, the population of *T. grandis* has decreased drastically in recent years, and was listed as a threatened species on the International Union for Conservation of Nature Red List (Yang & Luscombe, 2013). Additionally, *T. grandis* “Merrillii,” the only commercial cultivar, has been subjected to long-term planting, which has resulted in declined quality and yields (Li et al., 2004; Cheng et al., 2009). Therefore, the objective evaluation of wild resources is crucial for improving breeding programs and conservation of *T. grandis*.

The application of genetic information is often fundamental for the conservation and sustainable use of wild resources (Wayne & Morin, 2004; Hoban et al., 2013). The best way to develop strategies for the breeding and conservation of wild populations of *T. grandis* may be to obtain genomic information, thus revealing genetic diversity and population structure using molecular markers such as simple sequence repeats (SSRs). Due to the large and complex conifer genome, only a few conifer species have been subjected to genome sequencing (e.g., *Picea abies*, Nystedt et al., 2013; *Pinus taeda*, Neale et al., 2014). Alternatively, transcriptome sequencing by next-generation sequencing technologies, like expressed sequence tag (EST), is a promising strategy to obtain genomic information. Although a transcriptome have been generated for the cultivar *T. grandis* “Merrillii” that was selected by conventional breeding (Li et al., 2004; Yi et al., 2016), its limited genomic information might be insufficient for the investigation of wild genetic sources of *T. grandis*. Meanwhile, many studies on *T. grandis* have mainly focused on its medicinal component (Saeed et al., 2007), physiological ecology (Shen et al., 2014; Tang et al., 2015), nutrient content (Li et al., 2005; Feng et al., 2011), and field investigation (Li et al., 2005; Wu et al., 2006). Few studies have researched its genetic diversity. Thus far, a series of molecular markers were developed, but only amplified fragment length polymorphism and chloroplast microsatellites loci (cpSSR) have been applied to evaluate the genetic diversity of *T. grandis* (Min et al., 2009; Yi & Qiu, 2014; Zeng et al., 2014). However, these markers were dominant and/or uniparental inheritance, and underestimated the genetic diversity of *T. grandis*. Despite a set of codominant EST-SSRs developed from *T. grandis* “Merrillii” (Yi et al., 2016), these EST-SSRs were not employed to evaluate the genetic diversity and population structure of wild *T. grandis*.

In the present study, a comprehensive transcriptome from *T. grandis* leaves was obtained by Illumina sequencing platform, then the sequencing data were assembled and annotated, and a set of novel EST-SSR markers were developed from the transcriptome data. To effectively use and conserve wild *T. grandis*, the genetic diversity and population structure were also evaluated for six populations across its natural distribution using these EST-SSR markers.

## Materials and Methods

### Plant materials and DNA and RNA extraction

The fresh leaves of 84 individuals were collected from six wild populations of *T. grandis* across its natural distribution in China: 15 from Xinning, Hunan (XN), eight from Tonggu, Jiangxi (TG), 15 from Lichuan, Jiangxi (LC), 15 from Huangshan, Anhui (HS), 14 from Songyang, Zhejiang (SY), and 17 from Zhuji, Zhejiang (ZJ) (Fig. S1; Table S1). All sampled leaves were immediately preserved in silica gel. Total genomic DNA was extracted using a modified cetyltrimethylammonium bromide (CTAB) procedure (Doyle & Doyle, 1987). In addition, fresh young leaves of three different individuals from the ZJ population were rapidly frozen in liquid nitrogen after sampling and stored at −80 °C. Total RNA for these individuals were extracted using the TRIzol kit following the manufacturer’s procedures (Life Technologies, Carlsbad, CA, USA). The purity, concentration, and integrity of each RNA sample were determined using the NanoPhotometer spectrophotometer (IMPLEN, Westlake Village, CA, USA), the Qubit RNA Assay Kit in Qubit 2.0 Flurometer (Life Technologies, Carlsbad, CA, USA), and the RNA Nano 6000 Assay Kit of the Agilent Bioanalyzer 2100 system (Agilent Technologies, Santa Clara, CA, USA), respectively. Equal amounts of qualified RNA samples from each individual were pooled together for cDNA library construction and transcriptome sequencing.

### cDNA library construction and transcriptome sequencing

A three μg RNA sample was used for the RNA sample preparations. Sequencing libraries were generated using NEBNext Ultra RNA library Prep Kit for Illumina (New England BioLabs, Ipswich, MA, USA) following manufacturer’s recommendations. mRNA was purified using poly-T oligo-attached magnetic beads and sheared into short fragments using divalent cations under elevated temperature in NEBNext First Strand Synthesis Reaction Buffer (5×). First strand cDNA was synthesized using random hexamer primer and M-MuLV Reverse Transcriptase (RNaseH^−^). Second strand cDNA synthesis was subsequently performed using DNA Polymerase I and RNase H. Remaining overhangs were converted into blunt ends via exonuclease/polymerase activities. After adenylation of the 3′ ends of the DNA fragments, NEBNext Adaptors with hairpin loop structure were ligated to prepare for hybridization. In order to preferentially select cDNA fragments of 150–200 bp, the library fragments were purified with AMPure XP system (Beckman Coulter, Brea, IN, USA). Then three μl USER Enzyme (New England BioLabs, Ipswich, MA, USA) was used with size-selected, adaptor-ligated cDNA at 37 °C for 15 min, followed by 5 min at 95 °C before PCR. Then, PCR was performed with Phusion High-Fidelity DNA polymerase and universal PCR primers. Finally, PCR products were purified (AMPure XP system) and library quality was assessed on the Agilent Bioanalyzer 2100 system. The cDNA library was sequenced on the Illumina HiSeq 2500 platform (Illumina, San Diego, CA, USA) with 101-base paired-end reads at Novogene Biological Information Technology Co., Ltd., Beijing, China. The raw sequences of the transcriptome are available at figshare (https://doi.org/10.6084/m9.figshare.7006220.v2).

### Transcriptome assembly and functional annotation

After removing reads containing adaptor, reads containing more than 10% poly-*N*, and low-quality reads (>50% with Phred quality score (Q) ≤5 bases) from the raw sequencing data using in-house Perl scripts (Novogene Biological Information Technology Co., Ltd., Beijing, China), high-quality clean data were obtained and then assembled using Trinity software with min_kmer_cov set to 2 and all other parameters set to default (Grabherr et al., 2011). Briefly, the clean reads were firstly partitioned into many smaller pieces and these sequence data were assembled into contigs using the de Bruijn graph with k-mer 25 after a trial of different k-mer sizes. Next, the reads were mapped back to contigs with paired-end reads to detect contigs from the same transcript and measure the distances between these contigs. Finally, the contigs were linked to get assembled transcripts that could not be extended on either end. The longest transcripts were defined as the unigenes for gene functional annotation.

Gene function was annotated based on public protein databases: Nr (NCBI non-redundant protein sequences); Nt (NCBI non-redundant nucleotide sequences); KOG (Clusters of Orthologous Groups of proteins); Swiss-Prot (a manually annotated and reviewed protein sequence database) by NCBI blast 2.2.28+ with *e*-value 1.0*e*-5; Pfam (Protein family) by HMMER 3.0 package with *e*-value 0.01; and KO (KEGG Ortholog database) by KEGG Automatic Annotation Server with *e*-value 1.0*e*-10. Based on the annotated results of Nr and Pfam databases, the unigenes were assigned in Gene Ontology (GO) using Blast2GO 2.5 (Conesa et al., 2005) with *e*-value 1.0*e*-6. The distribution of GO functional classification was plotted using the Web Gene Ontology Annotation Plot (Ye et al., 2006) by three categories: biological process, molecular function, and cellular component.

### Identification of EST-SSRs

The MIcroSAtellite program (Thiel et al., 2003) was used to detect and locate EST-SSRs from unigenes. We set the detection criteria as at least six repeats for dinucleotide motifs, and five repeats for tri-, tetra-, penta-, and hexanucleotide motifs. Primer pairs flanking each EST-SSR were designed using PRIMER 3 (Untergasser et al., 2012), with PCR product size ranging from 80 to 300 bp, GC content between 40% and 60%, primer length ranging from 18 to 25 bp, and melting temperature between 55 and 65 °C.

PCR amplification was performed in a 10 μl reaction that included five μl sterile water, five μl 2 × *Taq* PCR MasterMix (Tiangen, Beijing, China), one μl of each primer (2.5 μM), and one μl 20–40 ng genomic DNA. The PCR procedure included an initial denaturation for 5 min at 94 °C, followed by 30 cycles of 40 s at 94 °C, 30 s at 55–65 °C for each locus, and 50 s at 72 °C, ending with a final extension of 7 min at 72 °C. The PCR products were initially detected using silver-stained non-denaturing polyacrylamide gels, then the polymorphic PCR products were sequenced by capillary electrophoresis using an ABI 3730 DNA Analyzer (Applied Biosystems, Foster City, CA, USA). Allele sizes were determined using GeneMarker version 2.2.0 (SoftGenetics, State College, PA, USA).

### Polymorphism of EST-SSR markers and population genetic analysis

To estimate the polymorphism of EST-SSR markers for each population of *T. grandis*, the genetic diversity indexes, number of alleles (*N*_A_), observed heterozygosity (*H*_O_), and expected heterozygosity (*H*_E_), were calculated using POPGENE version 1.32 (Yeh & Yang, 1999), polymorphism information content (PIC) was assessed using CERVUS 3.0.7 (Kalinowski, Taper & Marshall, 2007), and Hardy–Weinberg equilibrium (HWE) was detected using GenAlEx 6.5 with Chi-square tests (Peakall & Smouse, 2012). In addition, to infer the population genetic structure, Bayesian approach was implemented in STRUCTURE 2.3.4 (Pritchard, Stephens & Donnelly, 2000), assuming a population admixture model. The population genetic clusters (K) ranged from 1 to 6, and 20 independent runs were performed for each K with 20,000 burn-in and 20,000 Markov Chain Monte Carlo replicates. The most likely number K was estimated using ln*P*(*D*) (Pritchard, Stephens & Donnelly, 2000) and Δ*K* statistics (Evanno, Regnaut & Goudet, 2005). Based on inter-population genetic distances (*D*_A_) (Nei, Tajima & Tateno, 1983), the neighbor-joining (NJ) phylogenetic tree was constructed using POPTREE 2 (Takezaki, Nei & Tamura, 2010) with 1,000 replications for bootstrapping.

## Results

### Transcriptome sequencing and assembly

A total of 39,526,668 raw reads were generated from the Illumina sequencing of *T. grandis*. After removing adaptor-containing, more than 10% poly-*N*, and low-quality reads, 38,626,498 clean reads were obtained with 97.12% Q20 bases and 0.03% sequencing error rates. The total clean bases were 4.83 Gb with 43.54% GC content. Using the Trinity assembly software, the clean reads were assembled into 93,133 transcripts with mean length of 959 bp and N50 length of 1,838 bp. Of these, 64,356 (69.10%) transcripts ranged in length from 201 to 1,000 bp (Table 1). The transcripts were assembled into 69,920 unigenes, with mean length of 766 bp and N50 length of 1,503 bp. Of these, 54,564 (78.04%) transcripts ranged in length from 201 to 1,000 bp (Table 1).

**Table 1:** Length distribution of assembled transcripts and unigenes of *T. grandis*.

Length	Transcripts	Unigenes
201–300 bp	29,357	27,110
301–500 bp	19,347	16,529
501–1,000 bp	15,652	10,925
1,001–2,000 bp	15,692	8,698
>2,000 bp	13,085	6,658
Total	93,133	69,920
Minimum length (bp)	201	201
Mean length (bp)	959	766
Median length (bp)	465	364
Maximum length (bp)	12,196	12,196
N50 (bp)	1,838	1,503
Total nucleotides	89,285,608	53,541,465

### Functional annotation and classification

All of the 69,920 unigenes (100%) were successfully annotated in the seven public protein databases (Nr, Nt, KOG, Swiss-Prot, Pfam, KO, and GO). Of these unigenes, 23,140 (33.09%) were annotated in Nr, 8,686 (12.42%) in Nt, 8,914 (12.74%) in KOG, 17,591 (25.15%) in Swiss-Prot, 18,547 (26.52%) in Pfam, 7,797 (11.15%) in KO, 18,907 (27.04%) in GO, and 26,938 (38.52%) were annotated in at least one of the seven databases (Table S2).

**Figure 1:**
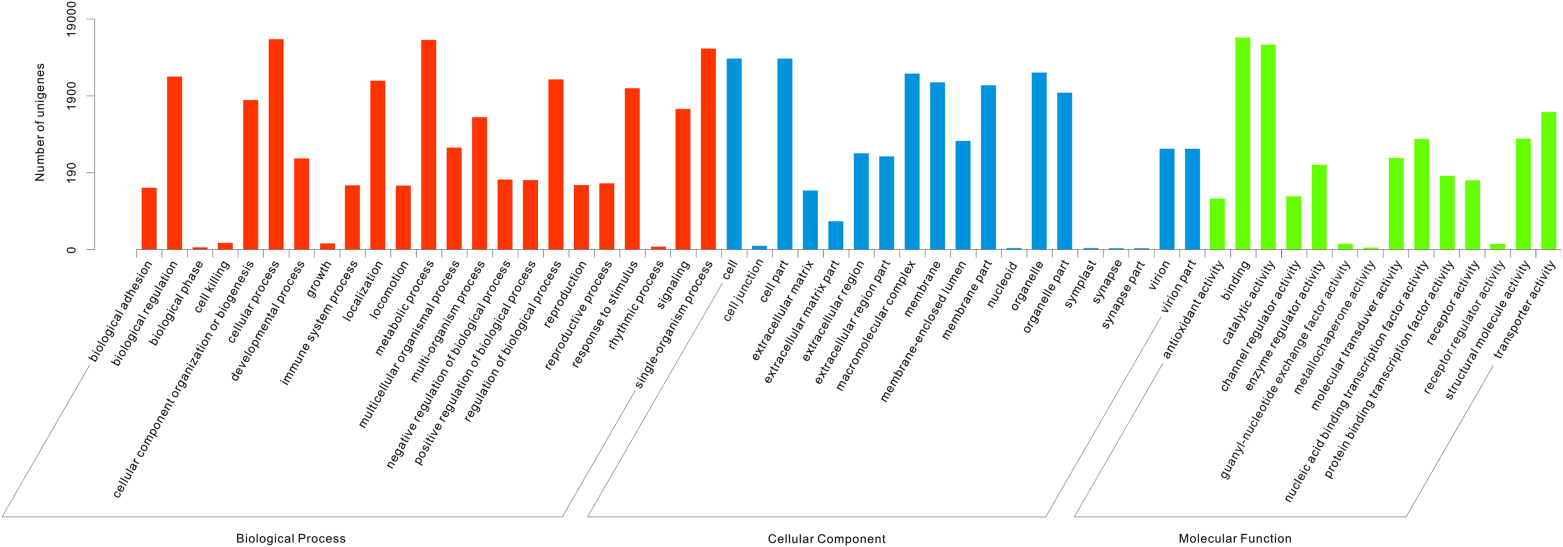
Functional classification of Gene Ontology (GO) for assembled unigenes of *T. grandis*. A total of 18,907 unigenes were assigned into 56 functional groups of three GO categories.

For functional classification of the assembled unigenes, 18,907 unigenes were assigned into 56 functional groups of three GO categories (Fig. 1). Of these unigenes, 11,587 were assigned into the biological process category, 5,807 into the molecular function category, and 6,990 into the cellular component category. In the biological process category, the most represented groups were “biological regulation” (3,362 unigenes), “cellular process” (10,355 unigenes), “metabolic process” (10,094 unigenes), and “single-organism process” (7,795 unigenes). Under the molecular function category, the most represented groups were “cell” (5,784 unigenes), “cell part” (5,782 unigenes), “macromolecular complex” (3,675 unigenes), and “organelle” (3,795 unigenes). Within the cellular component category, the most represented groups were “binding” (10,850 unigenes) and “catalytic activity” (8,791 unigenes) (Fig. 1).

All assembled unigenes were subjected to further functional prediction and classification by KOG and KO databases. There were 8,914 unigenes divided into 26 groups in the KOG database; the largest group was “General function prediction only” (1,902 unigenes), followed by “Posttranslational modification, protein turnover, chaperones” (1,135 unigenes) and “Signal transduction mechanisms” (743 unigenes) (Fig. S2). For KO analysis, 7,797 unigenes were assigned into 32 groups of five clusters; the largest group was “Signal transduction” (663 unigenes) of Environmental Information Processing cluster, followed by “Carbohydrate metabolism” (650 unigenes) of Metabolism cluster and “Translation” (642 unigenes) of Genetic Information Processing cluster (Fig. S3).

### Characterization of EST-SSRs and development of EST-SSR markers

**Figure 2:**
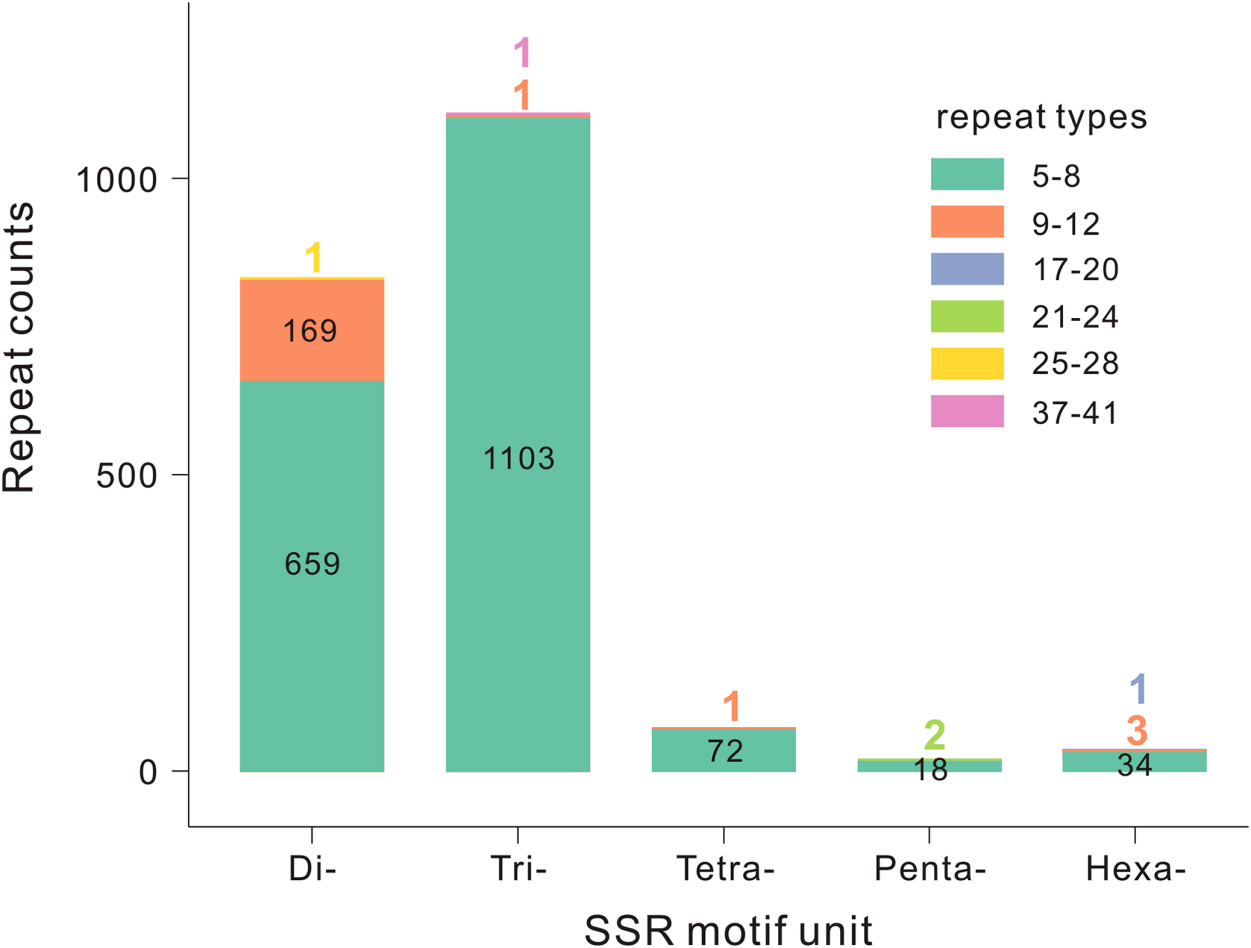
Distribution of SSR motifs and repeat types of *T. grandis*.

A total of 2,065 EST-SSRs were detected from 1,923 unigenes in the *T. grandis* transcriptome, with 158 unigenes containing more than one EST-SSR. Of the 2,065 EST-SSRs, 151 EST-SSRs were present in compound formation. The average number was 0.03 SSRs per unigene. Among all EST-SSR motifs, trinucleotide was the most common type (1,105, 53.51%), followed by dinucleotide (829, 40.15%), tetranucleotide (73, 3.54%), hexanucleotide (38, 1.85%), and pentanucleotide (20, 0.95%) (Fig. 2). The numbers of tandem repeats of these EST-SSRs ranged from five to 41, EST-SSRs with five to eight tandem repeats (1,886, 91.33%) were the most abundant, followed by nine to 12 tandem repeats (174, 8.43%), 17 to 28 tandem repeats (4, 0.19%), and 37 to 41 tandem repeats (1, 0.05%) (Fig. 2). Within these EST-SSRs, 84 motif types were identified. In the dinucleotide repeats, AT/AT (338, 16.37%) was the most abundant motif type, followed by AG/CT (276, 13.37%) and AC/GT (214, 10.36%). In the trinucleotide repeats, AAG/CTT (234, 11.33%) was the most abundant motif type, followed by AGG/CCT (224, 10.85%), AGC/CTG (185, 8.96%), AAT/ATT (138, 6.68%), and ATC/ATG (125, 6.05%). The remaining motif types accounted for 16.03% (Table S3).

Among the 2,065 EST-SSRs, 1,339 primer pairs were successfully designed, which mainly contained dinucleotide repeats (505, 37.72%) and trinucleotide repeats (723, 54.00%). From these primer pairs, 106 were randomly selected for amplification, and 31 had stable and clear PCR bands with expected size. The remaining primer pairs failed to yield PCR products, most produced multiple or dispersive bands. These 31 primer pairs were initially screened for polymorphisms, with 12 individuals from the six populations of *T. grandis*; 20 were monomorphic and 11 were polymorphic. The 11 polymorphic pairs were used for population genetic analysis (Table 2).

**Table 2:** Primer sequences, repeat motif, size range of alleles, and annealing temperature (*T*_m_) of 11 polymorphic EST-SSR markers developed for *T. grandis*.

Locus	Primer pair (5′–3′)	Repeat motif	Size range (bp)	*T*_m_ (°C)
GR12	F: GCTGTCGAAGCGTTGGAGAA	(ATTT)6	204–216	56
	R: TCTGAAACCTCGCTCGAACC			
GR16	F: GTCGCATGCACCTGTTCTTG	(TGCTA)5	140–150	57
	R: GCATAGCGGGGTGAATGAGT			
GR28	F: AATGGGAAATTTGGCAGGCG	(ACAGGC)8	290–314	56
	R: GGCCTTCACCACAGCTATGT			
GR29	F: CGTCAACGAGGGCAAAAAGG	(CAGAAC)9	122–152	56
	R: TTCTCTCCTACCGCCTTCGA			
GR34	F: GCTTGCGCGGATGTAAACAT	(GGGAGT)6	119–137	57
	R: GCGCAGAGCTTTCCAGTAAA			
GR44	F: TGATCTCAAGGGGGACTGCA	(GA)9	216–220	58
	R: CTTAGTATGCAGCCGAGGCA			
GR48	F: TTTTAGAACTGCTTGCCCGT	(CA)11	197–205	58
	R: CATGTACATGCACCATCATGC			
GR67	F: TCCAGTCAGCGCGAATAGTC	(TCC)12	141–162	58
	R: AGTAGAGGAGTCCATGGCGT			
GR80	F: AGCAGCATGGAGGACGATTC	(GCC)13	197–224	57
	R: CCTCGGGCATGTATCCATCC			
GR81	F: GGCTCAGTACTCCCAAACCC	(CCT)7	211–226	57
	R: TCGGCTCCTTTATACGACGC			
GR98	F: TATTCGAGACGCGCATTCGA	(ATCT)5	161–173	58
	R: CTCGCATTGAAGCTGTCTGC			

### Polymorphism of EST-SSR markers and population genetic diversity and structure

We estimated the polymorphism of 11 EST-SSR markers of *T. grandis*, and analyzed the population genetic diversity for six populations using these markers (Table 2). *N*_A_, PIC, *H*_O_, and *H*_E_ ranged from 1 to 6, 0 to 0.677, 0 to 1, and 0 to 0.752 for each locus, respectively. For each population, the mean *N*_A_, PIC, *H*_O_, and *H*_E_ ranged from 2.455 to 2.818, 0.322 to 0.389, 0.448 to 0.810, and 0.379 to 0.501, respectively. Of the 11 EST-SSR markers, GR81 significantly deviated from HWE in five populations apart from the TG population. GR48 significantly deviated from HWE in the HS and SY populations. GR16 significantly deviated from HWE in the HS population, while all markers, apart from GR44 and GR80, significantly deviated from HWE in the ZJ population (Table 3).

**Table 3:** Polymorphism of 11 EST-SSR markers and the Hardy–Weinberg equilibrium testing for each population of *T. grandis*.

Locus	XN (*N* = 15)					TG (*N* = 8)				
	*N*_A_	PIC	*H*_O_	*H*_E_	HWE *P*-value	*N*_A_	PIC	*H*_O_	*H*_E_	HWE *P*-value
GR12	2	0.371	0.467	0.508	0.8471 n.s.	3	0.447	0.625	0.575	0.8042 n.s.
GR16	2	0.164	0.2	0.186	0.6670 n.s.	1	0.359	0.5	0.5	0.8504 n.s.
GR28	2	0.164	0.2	0.186	0.6670 n.s.	1	0.258	0.125	0.325	0.0953 n.s.
GR29	2	0.239	0.333	0.287	0.4386 n.s.	1	0.354	0.143	0.495	0.0684 n.s.
GR34	1	0	0	0	n.a.	1	0	0	0	n.a.
GR44	2	0.239	0.333	0.287	0.4386 n.s.	1	0.215	0.286	0.264	0.6592 n.s.
GR48	4	0.566	0.933	0.653	0.0157 n.s.	6	0.636	1	0.742	0.5018 n.s.
GR67	3	0.443	0.769	0.542	0.1662 n.s.	3	0.456	0.5	0.592	0.7569 n.s.
GR80	4	0.61	0.733	0.69	0.3985 n.s.	1	0.195	0.25	0.233	0.6862 n.s.
GR81	3	0.541	1	0.638	0.0029*	3	0.544	1	0.658	0.0460 n.s.
GR98	4	0.655	0.571	0.73	0.4421 n.s.	3	0.398	0.5	0.492	0.3202 n.s.
Mean	2.636	0.363	0.504	0.428	–	2.455	0.351	0.448	0.443	–

	LC (*N* = 15)					HS (*N* = 15)				
	*N*_A_	PIC	*H*_O_	*H*_E_	HWE *P*-value	*N*_A_	PIC	*H*_O_	*H*_E_	HWE *P*-value
GR12	3	0.568	0.867	0.662	0.2797 n.s.	3	0.486	0.6	0.591	0.9639 n.s.
GR16	2	0.28	0.286	0.349	0.5708 n.s.	2	0.239	0.067	0.287	0.0032*
GR28	2	0.204	0.267	0.239	0.5513 n.s.	2	0.204	0.133	0.239	0.1013 n.s.
GR29	4	0.554	0.467	0.637	0.7777 n.s.	3	0.444	0.667	0.54	0.5406 n.s.
GR34	1	0	0	0	n.a.	2	0.062	0.067	0.067	0.8938 n.s.
GR44	2	0.117	0.133	0.129	0.7821 n.s.	2	0.062	0.067	0.067	0.8938 n.s.
GR48	4	0.51	1	0.616	0.0259 n.s.	3	0.517	1	0.618	0.0018*
GR67	3	0.24	0.286	0.265	0.9425 n.s.	3	0.529	0.714	0.622	0.6545 n.s.
GR80	3	0.198	0.231	0.219	0.9741 n.s.	4	0.361	0.467	0.402	0.9665 n.s.
GR81	3	0.504	1	0.612	0.0046*	3	0.492	1	0.6	0.0018*
GR98	4	0.677	0.8	0.752	0.4901 n.s.	4	0.658	0.714	0.738	0.7368 n.s.
Mean	2.818	0.35	0.485	0.407	–	2.818	0.369	0.5	0.434	–

	SY (*N* = 14)					ZJ (*N* = 17)				
	*N*_A_	PIC	*H*_O_	*H*_E_	HWE *P*-value	*N*_A_	PIC	*H*_O_	*H*_E_	HWE *P*-value
GR12	3	0.45	0.714	0.532	0.2288 n.s.	2	0.374	0.938	0.514	0.0004*
GR16	1	0.124	0.143	0.138	0.7735 n.s.	2	0.367	0.824	0.499	0.0039*
GR28	1	0.067	0.071	0.071	0.8898 n.s.	2	0.367	0.824	0.499	0.0039*
GR29	6	0.616	0.786	0.696	0.6323 n.s.	3	0.509	0.941	0.613	0.0003*
GR34	1	0	0	0	n.a.	2	0.367	0.824	0.499	0.0039*
GR44	3	0.325	0.429	0.37	0.7913 n.s.	3	0.406	0.813	0.522	0.0578 n.s.
GR48	1	0.375	1	0.519	0.0002*	3	0.54	1	0.633	0.0007*
GR67	3	0.385	0.546	0.45	0.6715 n.s.	2	0.366	0.813	0.498	0.0062*
GR80	1	0.067	0.071	0.071	0.8898 n.s.	2	0.105	0.118	0.114	0.7966 n.s.
GR81	3	0.541	1	0.638	0.0029*	3	0.416	1	0.544	0.0007*
GR98	6	0.59	0.643	0.683	0.8313 n.s.	3	0.457	0.813	0.575	0.0000*
Mean	2.636	0.322	0.491	0.379	–	2.455	0.389	0.81	0.501	–

**Notes:**

*N*, number of individuals; *N*_A_, number of alleles; PIC, polymorphism information content; *H*_O_, observed heterozygosity, *H*_E_, expected heterozygosity; HWE, Hardy–Weinberg equilibrium; n.s., not significant; n.a., Monomorphic locus.

* Deviated from HWE with a level of significance of *P* < 0.01.

**Figure 3:**
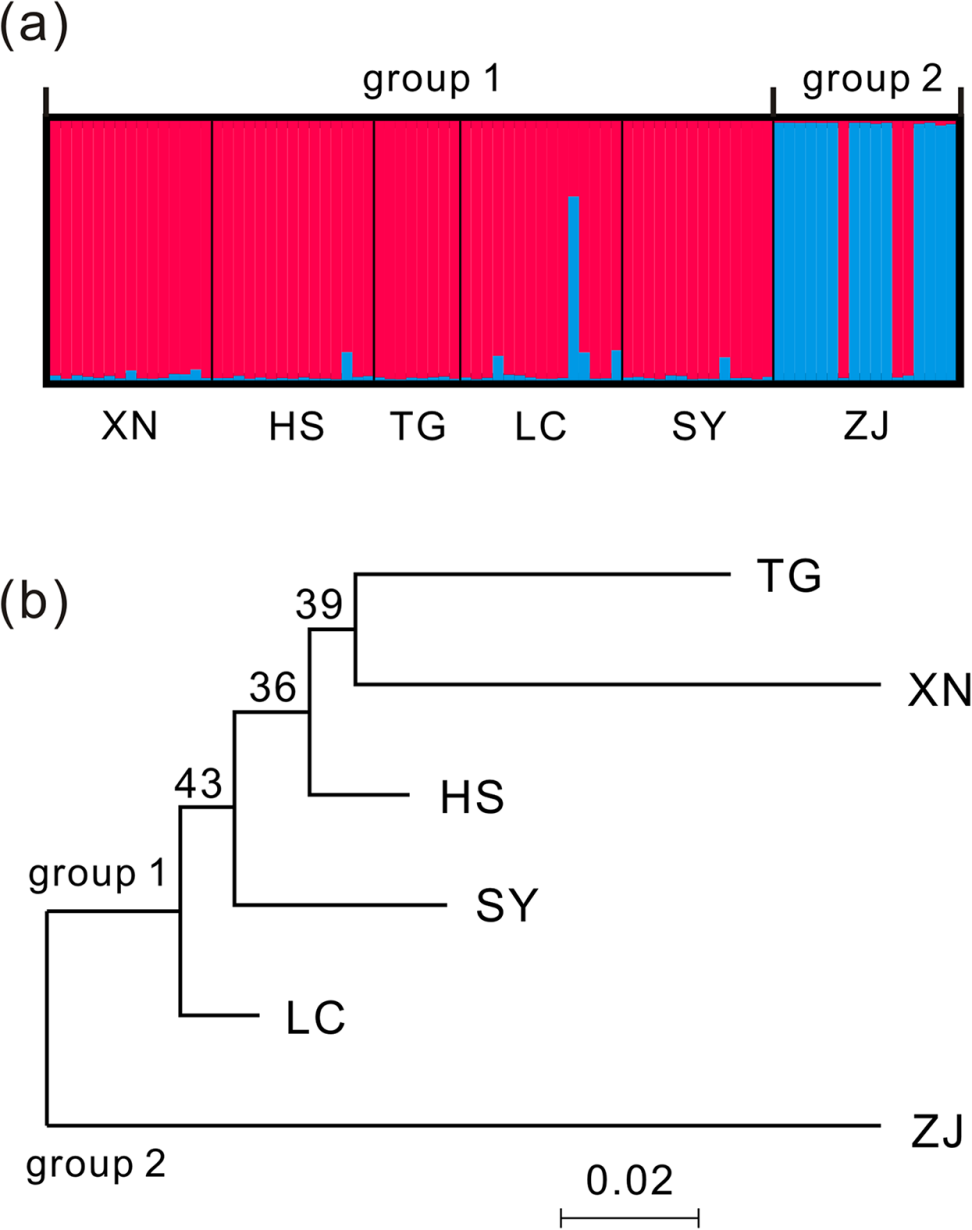
Two genetic groups of *T. grandis* identified by STRUCTURE analysis (*K* = 2) (A) and neighbor-joining (NJ) phylogenetic tree (bootstrap values above branches) (B). Six wild populations: XN, Xinning, Hunan; HS, Huangshan, Anhui; TG, Tonggu, Jiangxi; LC, Lichuan, Jiangxi; SY, Songyang, Zhejiang; ZJ, Zhuji, Zhejiang.

For the population structure of *T. grandis*, the most likely population genetic cluster K was estimated using ln*P*(*D*) and Δ*K* statistics with the STRUCTURE analysis. Because the ln*P*(*D*) increased gradually as K increased from 1 to 4, and did not show an apparent peak value, it was difficult to determine the most likely population genetic cluster. On the contrary, the Δ*K* statistic detected the highest peak at *K* = 2 (Fig. S4). The six populations of *T. grandis* were then assigned into two groups: Group 1 included the XN, TG, LC, HS, and SY populations; and Group 2 included only the ZJ population (Fig. 3A). Consistent with the STRUCTURE analysis, the NJ phylogenetic tree also revealed two groups-based *D*_A_ distances (Fig. 3B). The *D*_A_ distances between populations from Group 1 ranged from 0.041 to 0.137, while the *D*_A_ distances ranged from 0.151 to 0.229 between the ZJ (Group 2) population and the Group 1 populations (Table S4).

## Discussion

### Transcriptome characterization

Transcriptome sequencing is an effective and rapid approach to obtain genomic information for non-model plants (Ward, Ponnala & Weber, 2012; Strickler, Bombarely & Mueller, 2012), especially for conifer species with a large and complex genome (Morse et al., 2009; Zimin et al., 2014). In this study, a total of 38,626,498 clean reads (4.83 Gb) were generated from the transcriptome sequencing of *T. grandis*. The total number of assembled unigenes (69,920) was much larger than previously reported for *T. grandis* “Merrillii” (46,000) (Yi et al., 2016). Similarly, the mean and N50 length of the unigenes in our study, 766 and 1,503 bp (Table 1), respectively, were also longer than those reported for the cultivar (731 and 1,426 bp, respectively) (Yi et al., 2016), indicating that the transcriptome sequencing data were well assembled for *T. grandis* in the present study.

All of the unigenes identified in *T. grandis* were successfully annotated through seven protein databases (Nr, Nt, KOG, Swiss-Prot, Pfam, KO, and GO) (Table S2). The entire annotation for all unigenes was reported only in this study, but was not achieved for the other conifers, including *Pinus*, *Picea*, *Taxus*, *Cephalotaxus*, and *Sequoia* (Lorenz et al., 2012). It may suggest that the unigenes of *T. grandis* have more conserved functions. For example, complete genome sequencing revealed that the conifer species, *Picea abies*, has a large genome, but its number of functional genes is nearer to that of *Arabidopsis thaliana* (Nystedt et al., 2013). In fact, the success rates of functional annotation will be continually increased following the development of sequencing technologies and the abundance of protein databases. In addition, 18,907, 8,914, and 7,797 unigenes of *T. grandis* were assigned into 56 functional groups in GO category (Fig. 1), 26 in KOG (Fig. S2), and 32 in KO (Fig. S3), respectively, suggesting that these unigenes have various functions and will be valuable for functional gene discovery of *T. grandis*.

### Frequency and distribution of EST-SSRs

In this study, 1,923 unigenes containing 2,065 EST-SSRs were detected from total 69,920 unigenes in the transcriptome of *T. grandis.* The distribution frequency (2.75% of the total unigenes) and average number (0.03 SSRs per unigene) of EST-SSRs were lower than those reported for *T. grandis* “Merrillii” (3.72% and 0.037 SSRs) (Yi et al., 2016). The detection criteria of EST-SSRs may be generated by the difference between *T. grandis* and other conifer species. For example, in this study, a minimum of six, five, five, five, and five repeats were, respectively, used for di-, tri-, tetra-, penta-, and hexanucleotide motifs, whereas six, five, four, four, and four repeats were applied in *T. grandis* “Merrillii” (Yi et al., 2016), and six, five, five, four, and four repeats employed in *Pinus dabeshanensis* (Xiang et al., 2015). Therefore, the uniformly detected criteria of EST-SSRs are important and should be considered for the evaluation of distribution frequency and density of EST-SSRs.

In *T. grandis*, trinucleotide repeats were the most common type (53.51%), followed by dinucleotide repeats (40.15%), while tetra-, hexa-, and pentanucleotide repeats occupied a very small proportion (6.34%) (Fig. 2). This result was similar to previous reports that showed trinucleotide repeats were the most common type in *T. grandis* “Merrillii” (Yi et al., 2016) and other conifer species, such as *P. tabuliformis* (Niu et al., 2013), *P. dabeshanensis* (Xiang et al., 2015), and *P. pinaster* (Canales et al., 2014). Among these EST-SSRs, five to eight tandem repeats were the most abundant (91.33%) in *T. grandis* (Fig. 2), similar to *T. grandis* “Merrillii” (Yi et al., 2016). Moreover, the motif types of EST-SSRs in *T. grandis* were almost identical with those in the cultivar (Yi et al., 2016); AT/AT, AG/CT, and AC/GT contributed the most percentage for dinucleotide repeats, and AAG/CTT, AGG/CCT, AGC/CTG, AAT/ATT, and ATC/ATG contributed the most percentage for trinucleotide repeats (Table S3). Some of these motif types, such as AT, AG, AAG, AGG, AGC, and ATG, were also common in other conifer species (*Cryptomeria japonica*, Ueno et al., 2012; *P. halepensis*, Pinosio et al., 2014; *P. dabeshanensis*, Xiang et al., 2015; *P. taeda* and *P. abies*, Wegrzyn et al., 2014). This finding is likely a general case, suggesting that these motif types of EST-SSRs were relatively conservative in conifer species.

### Polymorphism of EST-SSR markers and population genetic diversity and structure

In the present study, 106 primer pairs of *T. grandis* were randomly selected from 1,339 primer pairs that were successfully designed for EST-SSR loci. Within these primer pairs, most of primer pairs (70.75%) yielded multiple or dispersive PCR bands, 31 (29.25%) had stable and clear PCR bands with expected size; the successful amplification was lower than that for *T. grandis* “Merrillii” (34.26%). Finally, 11 primer pairs (10.38%) were found with polymorphism (Tables 2 and 3), the success rate was also lower than that for the cultivar (17.59%) (Yi et al., 2016). Because the *Torreya* species are typical diploid plants (2*n* = 22) (Murray, 2013), the failed amplification that produced multiple or dispersive bands may be due to the highly repetitive sequences in conifer species (Kovach et al., 2010; Nystedt et al., 2013). Furthermore, *N*_A_ across six populations of *T. grandis* (2.636) was lower than that reported for the cultivar (3.059) (Yi et al., 2016), *C. japonica* (3.227) (Ueno et al., 2012), and *P. dabeshanensis* (3.000) (Xiang et al., 2015), while higher than that for *P. squamata* (2.200) (Mao et al., 2015). The average PIC of 0.357 was higher than that reported for *C. japonica* (0.325) (Ueno et al., 2012) and *P. squamata* (0.283) (Mao et al., 2015), while lower than that for *P. ponderosa* (0.618) (Lesser, Parchman & Buerkle, 2012) and *P. dabeshanensis* (0.380) (Xiang et al., 2015). In addition, the average *H*_O_ across six populations of *T. grandis* (0.540) was higher than that reported for *T. grandis* “Merrillii” (0.439), while the average *H*_E_ (0.432) was lower than that reported for the cultivar (0.522) (Yi et al., 2016). These results suggest that the polymorphism level of the 11 EST-SSR markers developed in this study is moderate in comparison with other conifer species and comparable to *T. grandis* “Merrillii” (Yi et al., 2016). Thus, the EST-SSR markers developed here, along with those previously developed, will be useful to investigate population genetic diversity and structure of *T. grandis*.

The STRUCTURE analysis and NJ phylogenetic tree revealed a distinct genetic population (ZJ) among the six populations of *T. grandis* (Fig. 3). Meanwhile, nine of the 11 EST-SSR markers developed in this study significantly deviated from HWE for the ZJ population, while one to three EST-SSR markers deviated from HWE for the remaining five populations (Table 3). A number of mechanisms could cause this disequilibrium and some are hard to be easily excluded, such as mutation, selection, and migration (Frankham, Ballou & Briscoe, 2010). But we prefer to attribute it to the introduction of some foreign genes. Because *Torreya* species are dioecious, wind-pollinated plants (Kang & Tang, 1995), gene introgression from related species into the population is possible. Moreover, *T. grandis* cultivation has been carried out for thousands of years in China and the ZJ population is located in Zhuji (Zhejiang, China) where is the main cultivation region of *T. grandis*, while cultivars are usually directly screened from wild populations (Li et al., 2004; Cheng et al., 2007), gene introgression from the cultivar could not have generated such a strong divergence among the *T. grandis* populations. Gene introgression from congeneric species, such as *T. jackii*, sympatric distribution with *T. grandis*, is more possible, because the two species have large genetic divergence; interspecific hybridization has been found between them, based on molecular and morphological evidence (Kou et al., 2017). In addition, it is also possible that the distinct genetic composition of the ZJ population came from another related species, *T. nucifera*, endemic to Japan. *T. nucifera* is the most closely related species with *T. grandis* in morphology and genetic relationship (Kou et al., 2017), and the species has records of cultivation because its seeds are also edible (Katsuki & Luscombe, 2013). However, this speculation needs to be verified by more population samples and further investigation of molecular markers.

## Conclusion

In summary, a whole transcriptome from the leaves of the conifer species *T. grandis* were produced by Illumina sequencing technology. A total of 69,920 unigenes were assembled, and all were annotated by searching against public protein databases. Across the transcriptome sequences, EST-SSRs (2,065) were detected and characterized with the distribution frequency of 2.75% of total unigenes and average number of 0.03 SSRs per unigene. Among these EST-SSRs, 1,339 primer pairs were successfully designed, and then 106 were randomly selected for the development of potential molecular markers. Of the 106 primer pairs, 11 polymorphic pairs, as the EST-SSR markers, showed a moderate level of genetic diversity (*N*_A_ = 2.636; PIC = 0.357; *H*_O_ = 0.540; *H*_E_ = 0.432). In addition, genetic structure and phylogenetic analysis revealed two different genetic groups, indicating that the set of EST-SSR markers developed in this study can be effectively applied to population genetic investigation, and may be used for genetic breeding and conservation of *T. grandis* in the future.

## Supplemental Information

10.7717/peerj.5606/supp-1 Supplemental Information 1 Fig. S1. Geographic distribution of six populations of *Torreya grandis* investigated in this study. The six populations of *T. grandis* were sampled across its natural distribution including Anhui, Zhejiang, Fujian, Jiangxi, Hunan provinces in China.

10.7717/peerj.5606/supp-2 Supplemental Information 2 Fig. S2. Functional classification of unigenes of *T. grandis* in KOG category. Total of 8,914 unigenes were assigned into 26 functional groups.

10.7717/peerj.5606/supp-3 Supplemental Information 3 Fig. S3. Functional classification of unigenes of *T. grandis* in KO category. Total of 7,797 unigenes were assigned into 32 functional groups of five clusters.

10.7717/peerj.5606/supp-4 Supplemental Information 4 Fig. S4. The most likely number of clusters (K) estimated with STRUCTURE analysis using ln*P*(*D*) (a) and Δ*K* (b) statistics.

10.7717/peerj.5606/supp-5 Supplemental Information 5 Table S1. Sampling details of six populations of *Torreya grandis*, included population cedes, collection locations, geographic coordinates, number of individuals.

10.7717/peerj.5606/supp-6 Supplemental Information 6 Table S2. Number and frequency of unigenes of *T. grandis* annotated in seven protein databases.

10.7717/peerj.5606/supp-7 Supplemental Information 7 Table S3. Number of motif types of EST-SSRs in *T. grandis*.

10.7717/peerj.5606/supp-8 Supplemental Information 8 Table S4. Genetic distances (*D*_A_) between each two of six populations of *T. grandis*.

10.7717/peerj.5606/supp-9 Supplemental Information 9 Raw data of Figure 3 and Table 3.

## Additional Information and Declarations

### Data Availability

The following information was supplied regarding data availability: The raw sequences of the transcriptome are available on figshare: Kou, Yixuan (2018): transcriptome data of *Torreya grandis*. figshare. Dataset. https://doi.org/10.6084/m9.figshare.7006220.v2 In addition, all supplementary data are available as Supplemental Files.
