# Supplementary figures and images for: Application of EST-SSR markers developed from the transcriptome of Torreya grandis (Taxaceae), a threatened nut-yielding conifer tree
Source: PeerJ. 2018 Sep 19;6:e5606. doi: 10.7717/peerj.5606 (PMC6151121; doi:10.7717/peerj.5606)

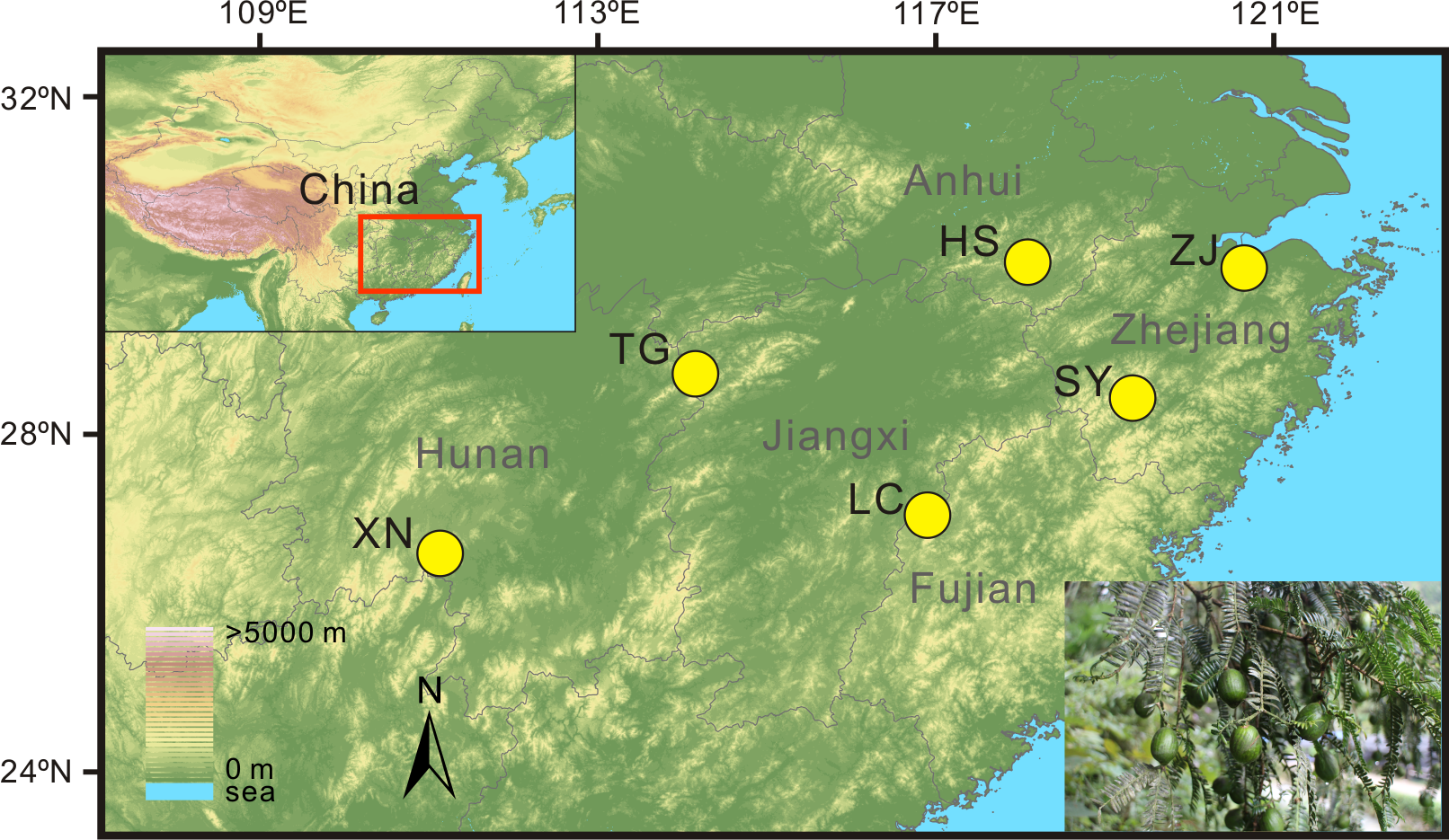

Supplement: Supplemental Information 1 — The six populations of T. grandis were sampled across its natural distribution including Anhui, Zhejiang, Fujian, Jiangxi, Hunan provinces in China. [file peerj-06-5606-s001.png]

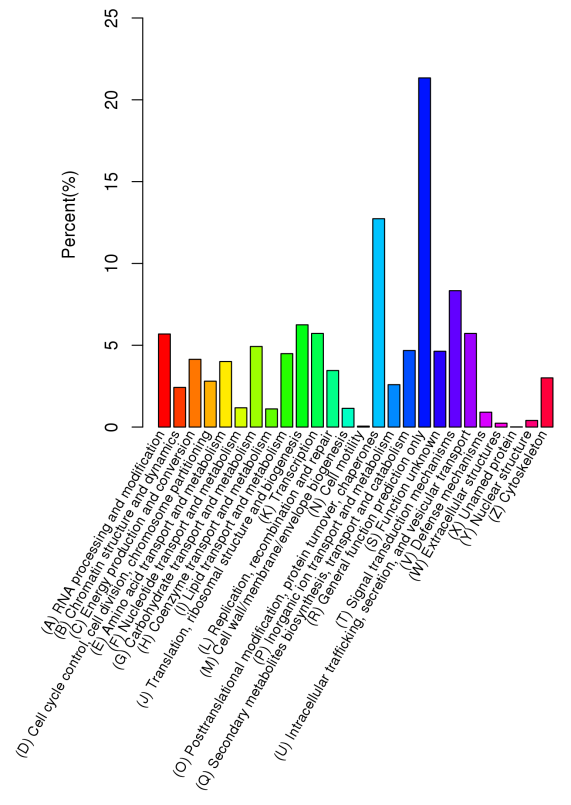

Supplement: Supplemental Information 2 — Total of 8,914 unigenes were assigned into 26 functional groups. [file peerj-06-5606-s002.png]

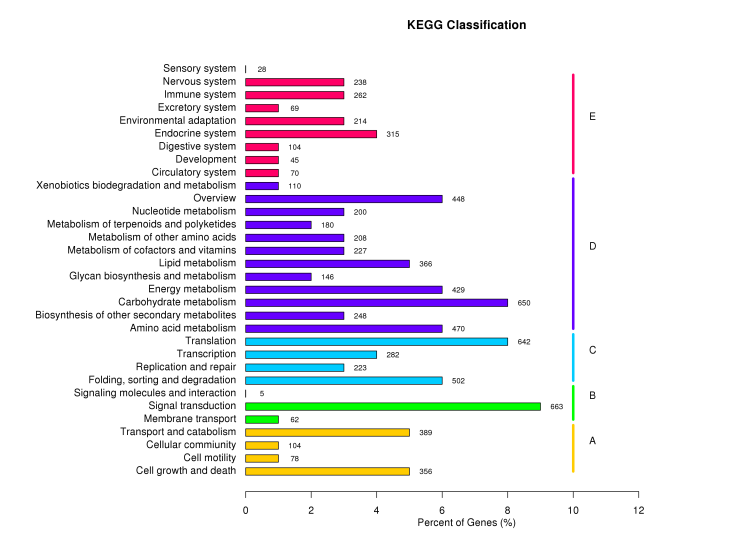

Supplement: Supplemental Information 3 — Total of 7,797 unigenes were assigned into 32 functional groups of five clusters. [file peerj-06-5606-s003.png]

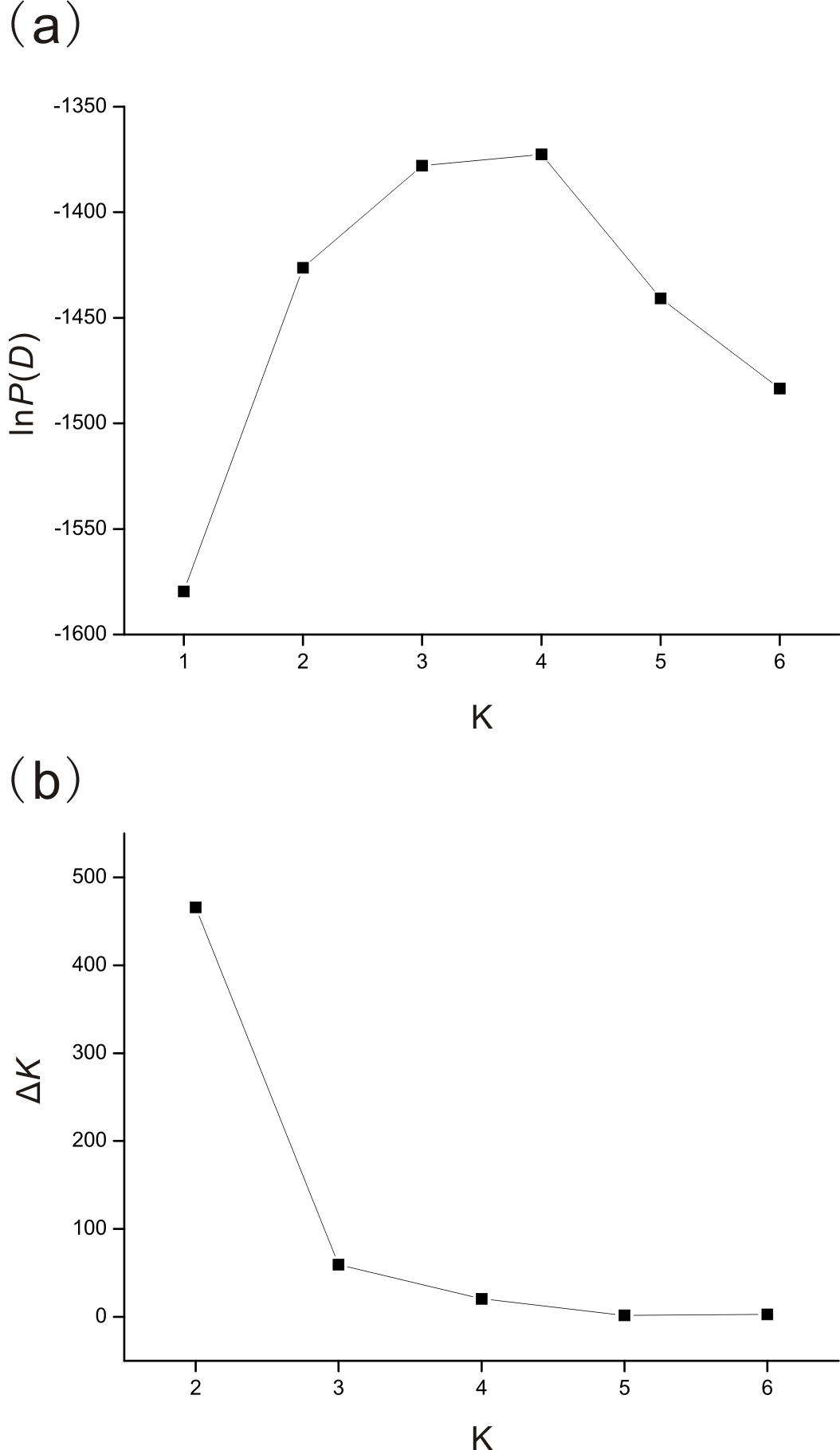

Supplement: Supplemental Information 4 [file peerj-06-5606-s004.png]
