# Supplementary material for: Application of EST-SSR markers developed from the transcriptome of Torreya grandis (Taxaceae), a threatened nut-yielding conifer tree
Source: PeerJ. 2018 Sep 19;6:e5606. doi: 10.7717/peerj.5606 (PMC6151121; doi:10.7717/peerj.5606)
Supplement: Supplemental Information 5 [file peerj-06-5606-s005.docx]

**Table S1** Sampling details of six populations of *Torreya grandis*, included population cedes, collection locations, geographic coordinates, number of individuals.

| Population code | Location | Latitude (N) | Longitude (E) | Altitude (m) | Individuals |
| --- | --- | --- | --- | --- | --- |
| XN | Xingning, Hunan | 26.569° | 111.163° | 461 | 15 |
| TG | Tonggu, Jiangxi | 28.702° | 114.185° | 346 | 8 |
| LC | Lichuan, Jiangxi | 27.038° | 116.921° | 586 | 15 |
| HS | Xiuning, Huangshan | 30.009° | 118.091° | 421 | 15 |
| SY | Songyang, Zhejiang | 28.361° | 119.324° | 357 | 14 |
| ZJ | Zhuji, Zhejiang | 29.697° | 120.519° | 364 | 17 |
