# Supplementary material for: Application of EST-SSR markers developed from the transcriptome of Torreya grandis (Taxaceae), a threatened nut-yielding conifer tree
Source: PeerJ. 2018 Sep 19;6:e5606. doi: 10.7717/peerj.5606 (PMC6151121; doi:10.7717/peerj.5606)
Supplement: Supplemental Information 6 [file peerj-06-5606-s006.docx]

**Table S2** Number and frequency of unigenes of *T. grandis* annotated in seven protein databases.

| Protein databases | Number of Unigenes | Percentage (%) |
| --- | --- | --- |
| Annotated in Nr | 23,140 | 33.09 |
| Annotated in Nt | 8,686 | 12.42 |
| Annotated in KOG/COG | 8,914 | 12.74 |
| Annotated in Swiss-Prot | 17,591 | 25.15 |
| Annotated in Pfam | 18,547 | 26.52 |
| Annotated in KO | 7,797 | 11.15 |
| Annotated in GO | 18,907 | 27.04 |
| Annotated in all Databases | 3,116 | 4.45 |
| Annotated in at least one Database | 26,938 | 38.52 |
| Total Unigenes | 69,920 | 100.00 |
