# Supplementary material for: Application of EST-SSR markers developed from the transcriptome of Torreya grandis (Taxaceae), a threatened nut-yielding conifer tree
Source: PeerJ. 2018 Sep 19;6:e5606. doi: 10.7717/peerj.5606 (PMC6151121; doi:10.7717/peerj.5606)
Supplement: Supplemental Information 7 [file peerj-06-5606-s007.docx]

**Table S3** Number of motif types of EST-SSRs in *T. grandis*.

| Repeats | 5 | 6 | 7 | 8 | 9 | 10 | 11 | 12 | 17 | 22 | 23 | 25 | 41 | Total |
| --- | --- | --- | --- | --- | --- | --- | --- | --- | --- | --- | --- | --- | --- | --- |
| AC/GT | – | 89 | 44 | 26 | 23 | 11 | 19 | 1 |  |  |  | 1 |  | **214** |
| AG/CT | – | 131 | 49 | 33 | 19 | 17 | 24 | 3 |  |  |  |  |  | **276** |
| AT/AT | – | 146 | 87 | 53 | 18 | 22 | 12 |  |  |  |  |  |  | **338** |
| CG/CG | – |  | 1 |  |  |  |  |  |  |  |  |  |  | 1 |
| AAC/GTT | 31 | 11 | 7 | 1 |  |  |  | 1 |  |  |  |  |  | 51 |
| AAG/CTT | 133 | 61 | 36 | 3 |  |  |  |  |  |  |  |  | 1 | **234** |
| AAT/ATT | 67 | 39 | 29 | 3 |  |  |  |  |  |  |  |  |  | **138** |
| ACC/GGT | 34 | 19 | 8 | 2 |  |  |  |  |  |  |  |  |  | 63 |
| ACG/CGT | 12 | 4 | 3 | 1 |  |  |  |  |  |  |  |  |  | 20 |
| ACT/AGT | 7 | 4 | 1 |  |  |  |  |  |  |  |  |  |  | 12 |
| AGC/CTG | 112 | 42 | 30 | 1 |  |  |  |  |  |  |  |  |  | **185** |
| AGG/CCT | 120 | 63 | 38 | 3 |  |  |  |  |  |  |  |  |  | **224** |
| ATC/ATG | 73 | 25 | 22 | 5 |  |  |  |  |  |  |  |  |  | **125** |
| CCG/CGG | 33 | 11 | 7 | 2 |  |  |  |  |  |  |  |  |  | 53 |
| AAAC/GTTT | 4 |  |  |  |  |  |  |  |  |  |  |  |  | 4 |
| AAAG/CTTT | 10 |  |  | 1 |  |  |  |  |  |  |  |  |  | 11 |
| AAAT/ATTT | 5 | 1 |  |  |  |  |  |  |  |  |  |  |  | 6 |
| AAGC/CTTG | 1 |  |  |  |  |  |  |  |  |  |  |  |  | 1 |
| AAGG/CCTT | 7 | 1 |  |  |  |  |  |  |  |  |  |  |  | 8 |
| AATC/ATTG | 1 |  |  |  |  |  |  |  |  |  |  |  |  | 1 |
| AATG/ATTC | 8 | 2 |  |  |  |  |  |  |  |  |  |  |  | 10 |
| ACAG/CTGT | 2 |  |  |  |  |  |  |  |  |  |  |  |  | 2 |
| ACAT/ATGT | 3 | 3 |  |  |  |  | 1 |  |  |  |  |  |  | 7 |
| ACTC/AGTG |  | 1 |  |  |  |  |  |  |  |  |  |  |  | 1 |
| AGAT/ATCT | 16 | 1 |  |  |  |  |  |  |  |  |  |  |  | 17 |
| AGCC/CTGG |  | 1 |  |  |  |  |  |  |  |  |  |  |  | 1 |
| AGGC/CCTG | 2 | 1 |  |  |  |  |  |  |  |  |  |  |  | 3 |
| ATCC/ATGG | 1 |  |  |  |  |  |  |  |  |  |  |  |  | 1 |
| AAACC/GGTTT | 1 |  |  |  |  |  |  |  |  |  |  |  |  | 1 |
| AAAGG/CCTTT |  |  | 1 |  |  |  |  |  |  |  |  |  |  | 1 |
| AACTC/AGTTG |  | 1 |  |  |  |  |  |  |  |  |  |  |  | 1 |
| AAGAT/ATCTT |  |  |  |  |  |  |  |  |  |  | 1 |  |  | 1 |
| AAGGC/CCTTG | 1 |  |  |  |  |  |  |  |  |  |  |  |  | 1 |
| AAGGG/CCCTT | 3 |  |  |  |  |  |  |  |  |  |  |  |  | 3 |
| AATAG/ATTCT |  |  |  |  |  |  |  |  |  | 1 |  |  |  | 1 |
| AATCC/ATTGG | 1 |  |  |  |  |  |  |  |  |  |  |  |  | 1 |
| AATTC/AATTG | 1 |  |  |  |  |  |  |  |  |  |  |  |  | 1 |
| ACAGT/ACTGT | 1 |  |  |  |  |  |  |  |  |  |  |  |  | 1 |
| ACCAG/CTGGT | 1 |  |  |  |  |  |  |  |  |  |  |  |  | 1 |
| ACCTC/AGGTG | 1 |  |  |  |  |  |  |  |  |  |  |  |  | 1 |
| ACTCC/AGTGG | 1 |  |  |  |  |  |  |  |  |  |  |  |  | 1 |
| ACTCG/AGTCG | 1 |  |  |  |  |  |  |  |  |  |  |  |  | 1 |
| AGAGC/CTCTG |  | 1 |  |  |  |  |  |  |  |  |  |  |  | 1 |
| AGCAT/ATGCT | 1 |  |  |  |  |  |  |  |  |  |  |  |  | 1 |
| AGCCC/CTGGG | 1 |  |  |  |  |  |  |  |  |  |  |  |  | 1 |
| ATCCG/ATCGG | 1 |  |  |  |  |  |  |  |  |  |  |  |  | 1 |
| AAAAAC/GTTTTT | 1 |  |  |  |  |  |  |  |  |  |  |  |  | 1 |
| AAAACC/GGTTTT | 1 |  |  |  |  |  |  |  |  |  |  |  |  | 1 |
| AAACTC/AGTTTG | 1 |  |  |  |  |  |  |  |  |  |  |  |  | 1 |
| AAATGG/ATTTCC | 1 |  |  |  |  |  |  |  |  |  |  |  |  | 1 |
| AACACC/GGTGTT |  |  |  |  |  | 1 |  |  |  |  |  |  |  | 1 |
| AACAGG/CCTGTT | 1 |  |  |  |  |  |  |  |  |  |  |  |  | 1 |
| AACCAC/GGTTGT | 1 |  |  |  |  |  |  |  |  |  |  |  |  | 1 |
| AACCAG/CTGGTT |  |  | 1 |  |  |  |  |  |  |  |  |  |  | 1 |
| AACCCT/AGGGTT |  |  |  |  | 1 |  |  |  |  |  |  |  |  | 1 |
| AACTGC/AGTTGC | 1 |  |  |  |  |  |  |  |  |  |  |  |  | 1 |
| AAGAGC/CTCTTG | 1 |  |  |  |  |  |  |  |  |  |  |  |  | 1 |
| AAGCCG/CGGCTT |  |  | 1 |  |  |  |  |  |  |  |  |  |  | 1 |
| AAGGCT/AGCCTT |  | 1 |  |  |  |  |  |  |  |  |  |  |  | 1 |
| AATACC/ATTGGT |  | 1 |  |  |  |  |  |  |  |  |  |  |  | 1 |
| AATACT/AGTATT |  |  |  | 1 |  |  |  |  |  |  |  |  |  | 1 |
| AATAGT/ACTATT |  | 1 |  |  |  |  |  |  |  |  |  |  |  | 1 |
| AATATG/ATATTC | 1 |  |  |  |  |  |  |  |  |  |  |  |  | 1 |
| AATCTG/AGATTC | 1 |  |  |  |  |  |  |  |  |  |  |  |  | 1 |
| AATGGC/ATTGCC |  | 1 |  |  |  |  |  |  |  |  |  |  |  | 1 |
| ACAGGC/CCTGTG |  | 1 |  |  |  |  |  |  |  |  |  |  |  | 1 |
| ACAGGG/CCCTGT |  | 1 |  |  |  |  |  |  |  |  |  |  |  | 1 |
| ACATAG/ATGTCT |  | 1 |  |  |  |  |  |  |  |  |  |  |  | 1 |
| ACATCC/ATGTGG | 1 |  |  |  |  |  |  |  |  |  |  |  |  | 1 |
| ACCCTC/AGGGTG |  | 1 |  |  |  |  |  |  |  |  |  |  |  | 1 |
| ACCTCC/AGGTGG | 1 |  |  |  |  |  |  |  |  |  |  |  |  | 1 |
| ACCTCT/AGAGGT |  |  |  |  |  |  |  |  | 1 |  |  |  |  | 1 |
| ACGAGG/CCTCGT | 1 |  |  |  |  |  |  |  |  |  |  |  |  | 1 |
| ACGCAG/CGTCTG | 1 |  |  |  |  |  |  |  |  |  |  |  |  | 1 |
| ACGGGG/CCCCGT | 1 |  |  |  |  |  |  |  |  |  |  |  |  | 1 |
| ACTCCC/AGTGGG | 1 |  |  |  |  |  |  |  |  |  |  |  |  | 1 |
| ACTCGG/AGTCCG |  | 1 |  |  |  |  |  |  |  |  |  |  |  | 1 |
| ACTGCT/AGCAGT | 1 |  |  |  |  |  |  |  |  |  |  |  |  | 1 |
| AGATGG/ATCTCC | 1 |  |  |  |  |  |  |  |  |  |  |  |  | 1 |
| AGCCAT/ATGGCT |  |  |  |  | 1 |  |  |  |  |  |  |  |  | 1 |
| AGCGGG/CCCGCT | 1 |  |  |  |  |  |  |  |  |  |  |  |  | 1 |
| AGGCGG/CCGCCT | 1 |  |  |  |  |  |  |  |  |  |  |  |  | 1 |
| ATCCCC/ATGGGG | 1 |  |  |  |  |  |  |  |  |  |  |  |  | 1 |
| ATCGGC/ATGCCG | 1 |  |  |  |  |  |  |  |  |  |  |  |  | 1 |
