# Supplementary material for: Application of EST-SSR markers developed from the transcriptome of Torreya grandis (Taxaceae), a threatened nut-yielding conifer tree
Source: PeerJ. 2018 Sep 19;6:e5606. doi: 10.7717/peerj.5606 (PMC6151121; doi:10.7717/peerj.5606)
Supplement: Supplemental Information 8 [file peerj-06-5606-s008.docx]

**Table S4** Genetic distances (*D*_A_) between each two of six populations of *T. grandis*.

| Population | TG | LC | HS | SY | ZJ |
| --- | --- | --- | --- | --- | --- |
| XN | 0.13 | 0.133 | 0.09 | 0.137 | 0.218 |
| TG |  | 0.077 | 0.081 | 0.094 | 0.229 |
| LC |  |  | 0.041 | 0.047 | 0.151 |
| HS |  |  |  | 0.053 | 0.183 |
| SY |  |  |  |  | 0.181 |
